# Why the *European Journal of Public Health* and EUPHA are opposing President Trump’s attack on the language of diversity

**DOI:** 10.1093/eurpub/ckaf018

**Published:** 2025-02-22

**Authors:** Peter Allebeck, Tit Albreht, Charlotte Marchandise, Martin McKee

**Affiliations:** European Journal of Public Health, Stockholm, Sweden; European Public Health Association, Utrecht, The Netherlands; European Public Health Association, Utrecht, The Netherlands; European Public Health Association, Utrecht, The Netherlands

The news that the Trump administration has ordered scientists at the US Centers for Disease Control and Prevention (CDC) to withdraw or retract articles containing terms such as “gender,” “transgender,” “LGBT,” or “transsexual” is as shocking as it is dangerous. The editors of the *British Medical Journal* (*BMJ*) have rightly condemned this act of political interference in scientific discourse [1]. Writing on behalf of the *European Journal of Public Health* and European Public Health Association (EUPHA), we stand alongside them in resisting these moves. Such censorship is not only an assault on scientific integrity but also a harbinger of the creeping authoritarianism that Europe has seen before, one that we must resist with all the force of history.

We in Europe know all too well the consequences of governmental overreach into scientific discourse. The 1930s were a period of intellectual darkness in much of our continent, as totalitarian regimes sought to purge academia and public health institutions of ideas they deemed “ideologically unsuitable.” In Nazi Germany, scientific research was subordinated to state ideology, with entire disciplines, such as genetics, psychology, and sociology, being restructured or erased to align with a doctrine of racial purity [2]. The Soviet Union under Stalin similarly purged scientists whose work did not conform to the dictates of Lysenkoism, leading to devastating consequences in agriculture and medicine [3].

The echoes of those policies are unmistakable in today’s America [4]. When a government instructs its scientists to withdraw papers that contain “forbidden terms,” it is engaging in ideological purging. This is not the way science works. Scientific inquiry thrives on open debate, the ability to ask difficult questions, and the freedom to publish findings based on empirical evidence, not political doctrine.

The ramifications of this directive extend far beyond the realm of academic publishing. The removal of gender-related data, the erasure of LGBT-related research, and the suppression of terminology essential for accurate scientific communication are not abstract concerns. They will harm real people. Gender and sexuality, as with other dimensions of diversity, are fundamental variables in public health research [5]. Ignoring them will result in poorer health outcomes for diverse populations, particularly those already marginalized.

Data underpins our work in public health. By studying how diseases affect different populations, whether based on sex, gender identity, ethnicity, or socioeconomic status, we can tailor interventions to those who need them most. A blanket suppression of terms does not change the underlying reality that gender and sexuality impact health. It simply makes it harder for policymakers, doctors, and researchers to address pressing health disparities. To excise such language is not just an act of censorship. It is an act of medical negligence.

The Trump administration’s move is part of a broader global trend in which political actors seek to undermine evidence-based policymaking. From climate change denial to vaccine misinformation, from attacks on reproductive rights to the rollback of gender protections, we see an alarming pattern: when science contradicts ideology, ideology attempts to silence science.

The European public health community must take this moment as a warning. The USA has long been a leader in medical research and public health innovation. If its institutions fall victim to authoritarian pressures, the repercussions will be felt worldwide. We have already seen European nations grapple with their own versions of this problem, whether in the restriction of reproductive rights in Poland, the rollback of LGBTQ+ protections in Hungary, or the rise of anti-science populism across the continent. No institution is immune if a world-leading public health agency like the CDC can be coerced into ideological conformity.

Our duty is to safeguard the integrity of the knowledge we produce and publish. The *BMJ* has taken a strong stance in refusing to comply with politically motivated retractions, and EUPHA and this journal will adhere to the same principles.

First, we will not retract published articles due to political pressure. Retractions are reserved for fraud, major errors, or ethical breaches, not for the mere use of words that a government disapproves of. Second, we will continue to publish research that includes terms related to gender, sexuality, and reproductive health. Third, we will defend the rights of researchers to publish without fear of political persecution. Fourth, we will stand in solidarity with colleagues facing censorship. Suppression of knowledge anywhere is a threat to knowledge everywhere.

History has shown us the cost of silence. The medical and scientific communities must act before this attack on knowledge spreads further. We call on all European public health institutions to formally denounce the CDC’s instructions and the Executive Order that gave rise to them and we urge researchers to speak out and resist any attempts to erase data or language essential to their work. We implore European politicians to ensure that our scientific institutions are free from political manipulation.

The lessons of the 1930s are clear. Censorship of science is never about protecting the public. It is about controlling them. When governments decide which words can and cannot be spoken and dictate what research can and cannot be published, they are not defending the truth. They are erasing it. We in Europe have seen where this road leads. We cannot allow history to repeat itself.

Conflict of interest: None declared.
